# Fanaticism

**Published:** 1859-06

**Authors:** 


### FANATICISM

Is seeing the seeming, as if it were real and acting accordingly; hence the fanatical merit our pity, instead of receiving our sneers, and our severer reprobation. In a radical sense, a fanatic is one who treats a phantom, a fancy, an apparition, a figment, as if it were a fact; and giving a wider scope, it is the exaggeration of a fact, or principle, or practice. It is on this latter that the success of many of the greatest enterprises of all ages have succeeded. A kind of fanaticism seems essential to any great success. It is a quality belonging to the ardent, to the highly imaginative, to the hopeful. But it may be well questioned, whether the world would not have made a steadier, a safer, and a farther progress, without the aid of this mental characteristic, with the advantage of having prevented the wasting of energies in a wrong direction, the blasting of highly cherished but unauthorized hopes, the utter ruin and wreck of many a fine intellect, the breaking of many a warm and noble heart.

In truth, fanaticism is a mental weakness ; it arises from an unbalancing of the faculties; an exaggeration of some, a deficit in others. Now and then the fanatical succeed ; but oftener, or at least more happily, do they succeed, who have what is called " well balanced minds. Such do not accomplish things as rapidly, but they do it with greater certainty, with greater durability, and with far less waste of power. In this equable adjustment of the high qualities, the English is a representative nation, while we find the type of the fanatical in the Frenchman ; the American is between the two.

As far as health and disease are concerned, we have instructive examples of the practical failure of fanaticism in the lives of Preisnitz, of Shew, of Graham, and Alcot. As citizens, all of them as far as we know were good men, honest, well meaning, benevolent and humane ; but when we look for the practical good effect of their theories, as exhibited in their own persons, and we may well suppose under the very highest advantages of correct, intelligent, and thorough application, there is confessedly a sad failure. Dr. Shew, the American champion of Hydropathy, died a comparatively young man. Preisnitz did not live to be old. Graham who gave name to the famous "Graham bread," died at the age of fifty, and Alcot, only completed his three score years; all of them frittered away their lives, in attempting to foist their crude notions upon public acceptance, with loud assurances of a serene and healthful old age. They exhibited great goodness of heart in their self denials, and their severe sacrifices, in attempting to prove the truth of their vagaries ; but this does not sanctify their own destruction, and the destruction of multitudes of weaker minded persons, who took hold of their half facts, and ran them into the ground, to their own undoing. Their sincerity, their honest belief in the truth of their theories, did not extend their own lives to an encouraging limit; while on the other hand, there is reason to suppose, they shortened them by their ill-advised experiments. Alcot drank no water for a whole year, and lived many years on fruits and vegetables ; never tasting meat, or milk, or butter, or yeasted bread, only to die at a time when both body and mind ought to have been in their highest prime.

Let these melancholy results learn us who still live, the true wisdom of avoiding extremes, remembering that a kind Providence has given us all things righty to enjoy, only enjoining to be temperate in the use of them, and in this, is enduring health, an effective life, a serene and happy old age.

